# Editorial

**Published:** 1868-10

**Authors:** 


### Editorial.

The regular course of Clinical instruction in the Mercy Hospital was resumed after the summer vacation, on the first Wednesday in September, and a class of good students have been regularly in attendance since. The regular clinics in the County Hospital commences on Friday, October 2d, and will be continued until next summer. The Rush Medical College commenced its regular annual lecture term on Wednesday, September 30th; and the course in the Chicago Medical College commences on Monday, October 5th. Chicago has become as truly a great centre for medical education, as for commerce.

In another part of the present number of the Examiner, we take the liberty to publish, entire, the Semi-Annual Report of the Surgical Institute at Jacksonville. In the paragraph relating to chronic ophthalmia, the author of the Report wishes a correction made, so that the second sentence shall read thus: "The division of the ciliary bands as practised by Mr. Hancock of London, and recommended by Dr. J. S. Hildreth of Chicago," etc.

Transactions of Ill. State Med. Society.—The annual volume of the Transactions of the Eighteenth Anniversary Meeting of the Illinois State Medical Society has been issued, and copies mailed to all the Members whose annual assessment for 1868 has been paid. If any fail to receive them they will please notify the Secretary, Dr. N. S. Davis, Chicago, Ill.

The present volume contains 112 pages, and is illustrated by several neatly executed cuts.

It contains reports on Chronic Inflammation of the Hip-joint, by Dr. R. G. Bogue, of Chicago; on Spinal Curvatures, by Dr. F. O. Earle, of Chicago; on Improved Form of Endoscope, by Dr. E. Andrews, of Chicago; on the Pathology and Treatment of Epidemic Cholera, by Dr. N. S. Davis, of Chicago; on Ophthalmology, by Dr. H. H. Roman, of Springfield, Ill.; on Obstetrics, by Dr. E. W. Moore, of Decatur, Ill.; and short papers by Drs. Holmes, of Chicago, E. P. Cook, of Mendota, and D. Prince, of Jacksonville.

---

Action of Caffein.—According to the experiments of M. Leven, as published in the *Archives of Physiology*, for Jan.–Feb., 1868, Caffein is a direct stimulant or excitant of nervous sensibility and action. Soon after its administration, the pulse is increased in frequency and force; respiration is accelerated; the muscular system contracts more vigorously, and secretions generally are moderately increased. It does not appear to be digested or appropriated in the system, but is readily elimenated within a few hours after it is taken.

---

Medical Depart. of the Cumberland University.—We have received a slip, by which we learn that a new Medical College with the above title has been organized in Nashville, Tenn.; and will commence its first regular course of instruction on the 2d of November next. *Prominent* as members of the Faculty, we notice the names of Paul F. Eve and Thomas R. Jennings, who recently retired from the faculty of the Medical Department of the University of Nashville, and E. S. Gaillard, late of the Richmond Medical College.

---

Louisville and Richmond Medical Journal.—A few months since, at the request of the Editor of the Journal just named, we inserted a paragraph stating that our subscribers could also be supplied with the *Louisville & Richmond Medical Journal* for $3,00 per annum. Since that time, the commutation arrangement has been discontinued, on account of the enlargement of that Journal. It is one of the largest and best monthly medical journals in the country, with a regular subscription price of $5,00 per annum.

---

Advertising.—We are often applied to by advertising agents and others for our terms of advertising, not only medical books, appropriate cards of pharmaceutists, druggists, etc., but all sorts of medical compounds, goods, wares, and merchandize.

We wish to inform all parties that the EXAMINER is intended to be a periodical devoted to medical science and education, and not a news board, with our name at the top, under which any-one can write their own puffs, or stick their own handbills, on the payment of a certain number of dollars. Our notions of professional obligation and propriety, will no more permit us to furnish the medium for heralding such advertisements as "Hunnewell's Eclectic Pills," "Hunnewell Tolu Anodyne," "Hygienic Wine," "Juniper Tar Soap," "Fourgera's Pectorial Paste," "Hoff's Malt Extract Beverage of Health," and a score of others, than to order them in prescriptions for our patients.

Neither will we encourage pharmaceutists to make up all sorts of combinations of medicines not officinally recognized in the pharmacopia. The combination of medicines adapted to the wants of each patient is one of the most delicate and important duties of the physician; requiring as much accuracy of judgment as the determining of the pathology or diagnosis of the case. The whole tendency of the use of such mixtures as "Elixir Calasaya, Iron, and Bismuth;" "Elixir Pepsine, Strychnia, and Bismuth," etc.; is to induce the practitioner to forego all adjustment of the quantity of each remedy to the special wants of each case. It equally tends to destroy all uniformity of the therapeutic experience.

If one practitioner uses the incongruous mixtures of one manufacturing house; another uses those of another house; and a third uses the medicines and formulas recognized as officinal, and each reports the results of his practice in a given disease, how are we to compare the results? Let pharmaceutists exercise their skill in giving us pure articles of medicine; in separating the active principles from the crude and inert parts; in making such combinations of the active principles as are truly chemical; and in putting the individual medicines into compact and palitable form for administration, and we will cordially aid them in their work. But let them leave the business of combining medicines designed for this or that disease or class of diseases entirely to the practitioner, at the bedside of his patient.

---

### Mortality Report for the Month of August:—

### CAUSES OF DEATH.

| | |
|---|---|
| Accident, drowned | 4 |
| " burned, | 2 |
| " fall of derrick | 1 |
| " machinery, | 1 |
| " railroad, | 3 |
| " shot, | 1 |
| " team, | 1 |
| " upsetting cart | 1 |
| Abdomen, injury of, | 1 |
| " contusion, | 1 |
| Angoria following scarlet fever, | 1 |
| Anæmia, | 1 |
| Apoplexy, | 3 |
| Apthæ, | 1 |
| Births, premature, | 26 |
| " still, | 28 |
| " tedious | 1 |
| Bowels, congestion of, | 2 |
| " inflammation, | 6 |
| Brain, congestion of, | 4 |
| " congestion of and organic disease of heart, | 1 |
| " congestion of and croup, | 1 |
| " congestion of and disease of spine, | 1 |
| " disease of, | 3 |
| " " " and cholera infantum, | 1 |
| " inflammation of, | 6 |
| Bronchitis, | 1 |
| " capillary, | 1 |
| " and diarrhœa | 1 |
| Breast cancer of, | 2 |
| Caries of spine and congestion of lungs, | 1 |
| Carbuncle, | 1 |
| Cellutitis pelvic, | 1 |
| Cholera infantum, | 266 |
| " " diarrhœa | 2 |
| " " enteritis | 1 |
| " " scarlet e | 1 |
| " " teething | 6 |
| " " whooping cough, | 4 |
| " morbus, | 9 |
| " " and diarrhœa | 1 |
| " " diptheria | 1 |
| " " rheumatism, | 1 |
| Colic, lead, | 1 |
| Childbirth | 1 |

| | |
|---|---|
| Convulsions, | 65 |
| " diarrhœa, | 3 |
| " enteritis | 1 |
| " teething | 5 |
| Croup, | 5 |
| " membranous | 1 |
| Cynanche maligna | 1 |
| Cyanosis, | 1 |
| Debility | 5 |
| Deficient development from injury | 1 |
| " vitality, | 1 |
| Diphtheria, | 1 |
| " and scarlet fever, | 1 |
| Diarrhœa, | 60 |
| " chronic, | 13 |
| " and dysentery | 1 |
| " convulsions | 1 |
| " diphtheria | 2 |
| Dropsy, | 3 |
| " abdominal, | 2 |
| " and general debility, | 1 |
| Duodenum, cancer of | 1 |
| Dysentery | 45 |
| " acute, | 2 |
| " and cholera morbus, | 1 |
| " gastritis, | 1 |
| " typhoid, | 1 |
| " teething | 2 |
| Dyspepsia | 1 |
| Endocarditis | 1 |
| Enteritis, | 8 |
| Encephalitis, | 5 |
| Enterocolitis | 10 |
| Epilepsy | 2 |
| Erysipelas, | 3 |
| " malignant | 1 |
| Exhaustion, | 1 |
| Fever, congestive, | 2 |
| " intermittent, | 2 |
| " puerperal, | 2 |
| " remittent | 1 |
| " scarlet, | 16 |
| " and angina, | 1 |
| " typhoid, | 29 |
| Gastritis | 1 |
| " chronic | 1 |
| " and diarrhœa, | 1 |
| " and whooping cough, | 1 |
| Gastrœntrites, | 5 |
| Gout | 1 |

| | |
|---|---|
| Hemorrhage, internal, | 2 |
| Hemoptysis, | 1 |
| Hepatitis, | 1 |
| Hernia strangulated | 1 |
| Heart, disease, | 3 |
| Heart, valvular disease | 1 |
| Hip, disease of | 1 |
| " contusion, | 1 |
| Hydrocephalus, | 16 |
| " chronic | 1 |
| " acute, | 4 |
| " chronic diarrhæa | 1 |
| " cholera infantum | 2 |
| " teething | 1 |
| Inanition, | 17 |
| Ileus, | 1 |
| Injuries by falling in well, | 1 |
| Jaundice | 5 |
| Laryngitis, | 2 |
| Liver, cirrhosis of | 1 |
| " congestion of, | 1 |
| Lungs, congestion of | 3 |
| " œdema of, | 1 |
| Measles, | 2 |
| Metral insufficiency | 1 |
| Meningitis | 7 |
| " convulsions | 1 |
| " diarrhœa, | 1 |
| " tuberculous | 1 |
| Mouth, canker sore of | 1 |
| Old age, | 5 |
| " chronic diarrhœa, | 1 |
| Opium, overdose, | 1 |
| Palate defective, | 1 |
| Parotitis and scarlet fever, | 2 |
| Paralysis, from sunstroke, | 1 |
| Peritonitis, | 6 |
| Pelvic and iliac cellular tissues suppuration of, from inflammation of caecum, | 1 |
| Pulmonum, ateleclasis | 2 |
| Pneumonia, | 10 |
| " and whoopingc-ough, | 1 |
| Phthisis, acute, | 1 |
| " pulmonalis | 41 |
| " rheumatism, | 1 |
| Phrenitis, | 1 |

Purpa and cholera infantum, ........ 1
Rheumatism, inflammatory, ........ 1
Stomach, cancer of, .. 2
" inflammation 1
" ulceration and hemorrhage - 1
" " from 1
Suicide, ........ 3

Skull, fracture of, .... 1
Tabes mesenterica, ... 39
Tetanus, ........ 1
Teething, ........ 37
" and diarrhœa 6
" hydrocephalus 1
Trismus ........ 1
Throat, cancer of, .... 1
Uræmia, ........ 1
Uterus, cancer of, .... 1

Unknown, ........ 2
Vitality, deficient, ... 1
Whooping-cough, .... 10
" " convulsions 1
" " diarrhœa, . 1
" " pneumonia 2
" " typhoid fever, ...... 1

Total, 943

COMPARISON.

Deaths in Aug., 1868, .. 943 | Deaths in Aug., 1867, .. 697 | Increase, .. 246

Deaths in July, 1868, ........ 897 | Increase, ........ 46

AGES.

| | | | | | |
|---|---|---|---|---|---|
| Under 5 | 733 | 40 to 50 | 41 | 90 to 100 | 0 |
| 5 to 10 | 25 | 50 to 60 | 12 | 100 to 110 | 1 |
| 10 to 20 | 21 | 60 to 70 | 20 | Unknown | 2 |
| 20 to 30 | 49 | 70 to 80 | 6 | | |
| 30 to 40 | 30 | 80 to 90 | 3 | Total | 943 |

| | | | | | |
|---|---|---|---|---|---|
| Males, | 511 | Females, | 432 | Total, | 943 |
| Single, | 841 | Married, | 102 | Total, | 943 |
| White, | 936 | Colored, | 7 | Total, | 943 |

NATIVITY.

| | | | | | |
|---|---|---|---|---|---|
| Chicago | 643 | England | 5 | Scotland | 4 |
| Other parts U. S. | 101 | Germany | 82 | Sweden | 15 |
| Austria, | 2 | Holland | 2 | Switzerland | 2 |
| Bohemia | 9 | Ireland | 45 | Unknown | 3 |
| Canada | 6 | Norway | 16 | Russia, | 1 |
| Denmark | 2 | Poland | 1 | | |
| Nova Scotia, | 1 | Wales, | 1 | Total, | 943 |
| New Foundland, | 1 | Italy, | 1 | | |

MORTALITY BY WARDS FOR THE MONTH.

| Ward. | Mortality. | Pop. in 1868. | One death in |
|---|---|---|---|
| 1 | 13 | 11,991 | 922 |
| 2 | 28 | 13,739 | 490 3-5 |
| 3 | 45 | 16,620 | 369 1-3 |
| 4 | 33 | 16,499 | 497 |
| 5 | 63 | 13,434 | 213 1-4 |
| 6 | 62 | 12,507 | 201 3-4 |
| 7 | 113 | 21,957 | 193 7-8 |
| 8 | 60 | 14,003 | 233 1-3 |
| 9 | 45 | 18,050 | 401 1-6 |
| 10 | 29 | 13,644 | 470 1-2 |
| 11 | 49 | 13,317 | 271 3-4 |
| 12 | 71 | 14,739 | 209 |
| 13 | 76 | 11,113 | 146 1-5 |

| Ward. | Mortality. | Pop. in 1868. | One death in |
|---|---|---|---|
| 14 | 70 | 14,168 | 202 1-3 |
| 15 | 72 | 20,429 | 280 1-3 |
| 16 | 41 | 16,011 | 393 |
| Bridewell, | 1 | | |
| County hosp. | 19 | | |
| Chi. River, | 2 | | |
| Mercy Hosp. | 4 | | |
| Police Stat. | 1 | | |
| Protes. Orphan asylum, | 2 | | |
| Lake Michi. | 3 | | |
| Immigrants | 34 | | |
| St. Joseph's Orph. As. | 1 | | |
| Home for Friendless, | 6 | | |

Total, ........ 943

Deaths.—The daily papers of St. Louis announce that on the 25th inst. (September) Joseph N. McDowell, M.D., of that city, died from congestive chills, aged 63 years.

Dr. McDowell had long occupied a prominent position, as a surgeon and teacher, in the profession. He was a man of great talents and remarkable eccentricities.

---

Clinical Lectures on Diseases of the Eye and Ear at the Chicago Eye and Ear Infirmary.—The Sixth Course of Lectures at the Infirmary, by Dr. E. L. Holmes, will commence on Monday, October 5th, at half-past 1 o'clock P.M., and will continue 20 weeks, on such days and at such hours as may be most convenient to those who attend.

No Institution in the North-West affords the student and practitioner superior opportunities for the clinical study of all forms of ophthalmic diseases, and their medical or surgical treatment.

During the past twelve months more than 800 patients received the benefits of the Institution.

During the last Course there was an average daily attendance of 40 patients, a large number of whom required important surgical operations.

Excellent opportunities will be afforded for studying each case and comparing it with other cases. The abnormal condition of the various tissues of the eye will be illustrated, not only by the cases under treatment, but also by numerous plates and a large number of pathological specimens.

The Tickets for the Course of 20 weeks will be $5.00 each. The fees will be devoted to the support of the Infirmary.

*Sept. 12, 1868, 16 East Pearson St.*

---

Money Receipts to Sept. 26th.—Drs. David B. Taylor, $3; H. L. Butterfield, 3; G. W. Rohr, 3; L. Tibbits, 3; W. H. Crawford, 1; T. D. Washburne, 3; W. H. Mussey, 12.

---

TO PHYSICIANS.—Prof. Horatio R. Storer will deliver his fourth private course of twelve lectures on the Treatment of the Surgical Diseases of Women, during the first fortnight of December, with illustrative operative instruction at the Franciscan Hospital for Women, under his charge.

Fee $50, and Diploma required to be shown. Certificates of attendance upon the previous courses have now been issued to twenty-nine gentlemen in different parts of the country.

*Hotel Pelham, Boston, Sept., 1868.*

---

CURABILITY OF CONSUMPTION. CASE OF PRESIDENT JEREMIAH DAY.—President Day, of Yale College, died in August, 1867, at the very advanced age of 95 years. He was an instance of what care and judicious treatment can effect in a disease often considered necessarily fatal. We take some of the facts of his life, and of the autopsy, from the *Trans. of the Connecticut State Medical Society*, reported by Professor S. G. Hubbard, M.D., of New Haven.

Jeremiah Day was born in Washington, Conn., August 2d, 1773; and during the war of Independence was old enough to appreciate the nature of the issue involved in that struggle, and well remembered having seen some of the principal actors in it.

His infancy and boyhood were marked by indications of feeble vitality; and the prospect of his arriving to the maturity of manhood, never very flattering, sensibly diminished as he approached that period. He entered the Freshman class in Yale College in 1789, but was soon obliged to leave college on account of a "pulmonary difficulty," which was, doubtless, the incipient stage of the organic disease of the lungs, which subsequently developed itself. These symptoms were so far alleviated, that for two years he taught a school in Kent and Winchester, when he found his health so much improved that he returned to college, and was graduated in the class of 1795.

The succeeding six years, a period of great feebleness, were spent partly in teaching at Greenfield for a year, as tutor in William's College for two years, and as tutor in Yale College for three years, during which last period he studied Theology, and preached occasionally in vacant churches in the vicinity, until 1801, when he was elected Professor of Mathematics and Natural Philosophy in the College.

He was prevented, however, from entering upon his professional studies, by the occurrence of an alarming pulmonary hemorrhage, which happened after a Sabbath service at West Haven, where he preached for the Rev. Dr. Williston. Other hemorrhages followed, by which he was greatly prostrated, losing large quantities of blood. According to the prevailing practice at that time, he was freely bled from the arm—"the doctors taking," as he remarked to me, "nearly all of the little remaining blood in his body."

In this condition of extreme exhaustion, at the age of twenty-eight, he abandoned temporarily the purpose of entering upon the duties of his professorship, and in September of that year, he made a voyage to Bermuda, to try the effect upon his health of warm climate. While there he was treated with tincture digitalis to the extent of producing its culmulative effects, which were so profoundly sedative, that for a time his life was despaired of. Indeed so reduced and attenuated was he on leaving home, that none of his friends expected to see him again alive, and the published letters of Professor Kingsley and others of that period, lament him as already lost to science and the world. He returned, however, in the following April, but without having experienced any material benefit; so that he now gave up all idea of fulfilling his collegiate appointment; and bidding farewell to his associates, he retired to his home among the hills of Washington, to die.

The hemorrhages continued, and were followed by venesections, until a Dr. Sheldon, of Litchfield, who enjoyed a wide reputation for "curing consumption," chanced to see him, and causually remarked that he needed Iron,"—and "he believed he could cure him."

Although the patient was evidently in a hopeless decline, he was placed under the care of Dr. Sheldon, who would seem to have been an acute observer, and in his knowledge of pathology and therapeutics, far in advance of his time. Under the use of preparations of iron with bark, and nutritious food, Mr. Day soon began to exhibit signs of returning strength and health; and in 1803, although he seemed to his friends literally like one raised from the dead, he was so far restored to health, as to be inaugurated as professor. From this time all symptoms of pulmonary disease disappeared, and did not return.

On opening the thorax, only a moderate quantity, perhaps a pint, of serum was found in both cavities,—the lungs were every where quite free from tubercular deposit, and in all respects healthy. In the apex of each lung, however, was found a dense, corrugated circular cicatrix, an inch and a half or more in diameter,—also a *third* cicatrix, on the left side of the left lung, a few inches below the apex, each involving such a depth of tissue, as to indicate that the vomicæ of which they were the remains, had been large and of long duration. Both lungs were slightly adherent at the apex.

Here then, was all that remained to mark the beginning, progress, and cure of a case of tubercular consumption, occupying *twelve years* in its period of activity, and with its incipient stage, dating back more than *three-quarters of a century*. A legible record, surpassing in interest and importance to the human race, those of the slabs of Nineveh, or the Runic inscriptions. —*Med. and Surg. Reporter*.

---

The Trustees of the Fiske Fund, at the annual meeting of the Rhode Island Medical Society, held in Providence, June 10, 1868, gave notice that no awards had been made on the questions proposed by them the past year.

They offer the following subjects for 1868:

1. Bromides, their physiological effects and therapeutical uses.

2. Cerebro-Spinal Meningitis, pathology and treatment.

3. "Grave's disease" (so called), pathology and treatment.

4. Carbolic Acid, its therapeutical effects and hygienic uses.

For the best dissertation on each of these subjects they offer a premium of one hundred dollars.

Every competitor for a premium is expected to conform to the following regulations, *viz.:*

To forward to the secretary of the Fiske Fund Trustees, on or before the first day of May, 1869, free of all expense, a copy of his dissertation, with a motto written thereupon, and also accompanying a sealed packet, having the same motto inscribed upon the outside, and his name and place of residence within.

Previously to receiving the premium awarded, the author of the successful dissertation must transfer to the trustees all his right, title, and interest in and to the same, for the use, benefit, and behoof of the Fiske Fund.

Letters accompanying the unsuccessful dissertations will be destroyed by the trustees, unopened, and the dissertations may be procured by their respective authors, if application be made therefor within three months.

Address,

S. Aug. Arnold, M.D., Providence,
Secretary of Fiske Fund Trustees.

---

At the annual meeting of the Committee on the Boylston Medical Prize Questions, on the first Wednesday in June, 1868, it was announced that no dissertation had been presented on either of the questions proposed.

The following questions are proposed for 1869:

1. Food in Disease, acute and chronic; its variety, advantages, dangers, and relation to appetite.

2. The Surgical Treatment of Hemorrhoids, and the Surgical Treatment of Fistula in Ano, with its result.

The author of the best dissertation on either of the subjects proposed for 1869 will be entitled to a premium of one hundred and fifty dollars.

Dissertations on these subjects must be transmitted, post-paid, to John Jeffries, M.D., on or before the first Wednesday in April, 1869.

The following are the questions proposed for 1870:

1. The Modern Pathology of Tumors.

2. Aphasia, or the Relation of the Brain to Speech.

Dissertations on these subjects must be transmitted as above, on or before the first Wednesday in April, 1870.

The author of the best dissertation considered worthy of a prize, on either of the subjects proposed for 1870, will be entitled to a premium of two hundred dollars.

Each dissertation must be accompanied by a sealed packet, on which shall be written some device or sentence, and within which shall be enclosed the author's name and residence. The same device or sentence is to be written on the dissertation to which the packet is attached.

The writer of each dissertation is expected to transmit his communication to the President, John Jeffries, M.D., in a legible handwriting, within the time specified.

---

M. Nelaton.—The elevation of this eminent surgeon to a Senatorship by Napoleon III. has excited more than usual interest, inasmuch as it was stated to be the more honorable, not being accompanied by the condition that he should retire from the practice of his profession, as was the case when the same honor was offered to M. Double by Louis Philippe. It would appear, from the following statement by M. Latour (*Union Medicale*), that there is some doubt about this matter; and he gives a correct version of the anecdote about Double. Double was the friend and physician of Marshal Soult, and it was at his request that Louis Philippe consented to create Double a peer, on the condition, however, that he give up practice. The Marshal took the nomination to Double himself, and stated the condition attached to it. Double was very rich, approaching the end of his career, of simple, or even austere, habits; and certainly the desire of gain, and of the enjoyments obtainable by money, had no part in the resolution he came to. "Tell the King," he replied to the Marshal, "I can submit to no conditions. If I am to enter the Luxembourg, it must be stethoscope in hand." This was too much for Louis Philippe, and the affair came to nothing. After all that has been said about the greater liberality manifested towards M. Nélaton by attaching no such conditions, it seems that he is to retire from practice! It is true that he has some time contemplated doing this, and has been withdrawing from consultations and operations; but it is very doubtful whether he would have received the nomination had this not been known to be the case. With respect to the immense income he is said to derive from his practice, M. Latour believes the statement that it amounts to 600,000 francs is a great exaggeration. Trousseau, at his most flourishing period, observed that there were but three practitioners in Paris whose income had sometimes exceeded 200,000 francs —*viz.:* himself, and MM. Nélaton and Ricord. However this may be, Nélaton's retirement, and the deaths within so short a space of time of men so largely consulted as Trousseau, Velpeau, Rayer, and Jobert, have placed immense opportunities within the reach of the rising practitioners.

---

The Physician's Visiting List.—This annual volume, issued by Messrs. Lindsay & Blakiston, has now reached the 18th year of its publication, and is so well known, and its usefulness is so generally recognized, that it is sufficient to announce that the List for 1869 has appeared, and may be obtained from all booksellers.—*Ibid.*

